# Lateral movement of the saddle relative to the equine spine in rising and sitting trot on a treadmill

**DOI:** 10.1371/journal.pone.0200534

**Published:** 2018-07-18

**Authors:** A. Byström, L. Roepstorff, M. Rhodin, F. Serra Bragança, M. T. Engell, E. Hernlund, E. Persson-Sjödin, R. van Weeren, M. A. Weishaupt, A. Egenvall

**Affiliations:** 1 Department of Anatomy, Physiology and Biochemistry, Faculty of Veterinary Medicine and Animal Science, Swedish University of Agricultural Sciences, Uppsala, Sweden; 2 Department of Clinical Sciences, Faculty of Veterinary Medicine and Animal Science, Swedish University of Agricultural Sciences, Uppsala, Sweden; 3 Department of Equine Sciences, Faculty of Veterinary Medicine, Utrecht University, Utrecht, The Netherlands; 4 Equine Department, Vetsuisse Faculty University of Zurich, Zurich, Switzerland; Liverpool John Moores University, UNITED KINGDOM

## Abstract

Saddle slip, defined as a progressive lateral displacement of the saddle during ridden exercise, has recently been given attention in the scientific press as a potential sign of lameness. The aim of this study was to objectively quantify the normal lateral movement (oscillations) of the saddle relative to the horse in non-lame horses, and associate this movement to the movements of the horse and rider. Data from seven Warmblood dressage horses competing at Grand Prix (n = 6) or FEI Intermediate (n = 1) level, ridden by their usual riders, were used. Simultaneous kinetic, kinematic and saddle pressure measurements were conducted during sitting and rising trot on a force-measuring treadmill. The maximum lateral movement of the caudal part of the saddle relative to the horse's spine (MAX) was determined for each diagonal step. A mixed model was applied, with MAX as outcome, and T6 and S3 vertical position, rigid body rotation angles (roll, pitch, yaw) of the horse’s and rider’s pelvis, vertical ground reaction forces, saddle force, and rider position (rising in rising trot, sitting in rising trot or sitting in sitting trot) as explanatory variables. The least square means for MAX were 14.3 (SE 4.7) mm and 23.9 (SE 4.7) mm for rising and sitting in rising trot, and 20.3 (SE 4.7) mm for sitting trot. A 10 mm increase in maximum pelvic height at push off increased MAX by 1.4 mm (p<0.0001). One degree increase in rider pelvis roll decreased MAX 1.1 mm, and one degree increase in rider pelvis yaw increased MAX 0.7 mm (both p<0.0001). The linear relationships found between MAX and movements of both horse and rider implies that both horse and rider movement asymmetries are reflected in the lateral movements or oscillations of the saddle in non-lame horses.

## Introduction

Saddle slip, described as a progressive displacement of the saddle from the horse’s midline during ridden exercise [1], has recently been a topic of study in several scientific publications. In a population of 506 sport horses, which were in regular use and deemed sound by their owners, the saddle slipped consistently to one side in 62 horses [2]. In a series of 128 horses presented for clinical gait assessment, the saddle consistently slipped to one side in 39 horses, and saddle slip was abolished by resolution of hindlimb lameness through diagnostic anesthesia in 37 of these cases [1]. In the latter cases the saddle slipped towards the lamer hindlimb side in 32 of the 37 cases. Saddle slip associated with lameness was most often present on one rein only, and horses with a wider (more barrel-like) back profile were more prone to saddle slip [2–3]. These same studies found that crookedness of the rider and the fit of the saddle were independently associated with saddle slip in lame and non-lame horses, but the authors argue that these factors cause saddle slip mainly in interaction with hind limb lameness, rather than being causal factors per se.

The abovementioned studies on saddle slip are all clinical and observational in character. Using quantitative motion analysis, the kinematics of the saddle itself have been studied in ridden and unridden horses [4–6], but on the movements of the saddle relative to the horse there is only a single study at walk [7]. Consequently, current knowledge about the effects of various factors on the movements of the saddle relative to the horse is mainly based on practical clinical experience and limited empirical evidence. Therefore, more quantitative and objective studies on the issue are needed, especially if clinical inferences are based on the presence of saddle slip.

In this study we used material from a previous study in which synchronized data on ground reaction forces, kinematics and saddle pressure were collected from seven high-level dressage horse- and rider combinations walking and trotting on a treadmill [4,7–9]. This data set was deemed to be highly suited for obtaining better insights in the complex interaction between horse, rider, and saddle movements, complementary to the observational and subjective knowledge currently available. The aims of this study were to objectively quantify the normal lateral movement (oscillations) of the saddle relative to the horse's back in non-lame horses during steady state, straight line trot, and to evaluate associations between the magnitude and direction of this movement and a selection of kinetic and kinematic variables representing the movements of the horse and rider. We included trials in sitting trot as well as left and right rising trot, since rising trot is known to cause rider-induced asymmetries in the horse's movement pattern [10–11] and saddle slip has often been evaluated during rising trot [1].

## Materials and methods

### Horses and riders

Seven dressage horses competing at Grand Prix (n = 6) or Fédération Equestre International (FEI) Intermediate (n = 1) level participated in the study. The horses were of Warmblood breed (withers height range 1.64–1.85 m), and equipped with their own fitted dressage saddle and a bridle with a normal snaffle bit. Before the experiment, the saddles were inspected by one person in the research team experienced in saddle fitting. Based on manual palpation and pressure distributions obtained from the saddle pressure mat, the saddles were deemed to fit appropriately. Horses were ridden by their usual riders, three males and four females (body mass 78 (SD±17) kg). Before the measurements the horses were checked for soundness by an experienced veterinarian (assessed in hand on hard ground) and were found to be free of lameness and dysfunction of the back. This study protocol was approved by the Animal Health and Welfare Commission of the canton of Zürich (188/2005).

### Design

The study set-up has been previously described [8–9]. For the current analysis, data from sitting trot and left and right rising trot (rider rising on the left and right hind limb diagonal, respectively) trials in one head and neck position (the frontal contour of the horse’s head aligned with the vertical) were used. There were several sitting trials available and the trial at the speed closest to the rising trials was chosen for each horse. Data were collected for 15 s for four horses and for 12 s for three horses (due to technical reasons).

### Technical equipment

Saddle pressure distribution was measured with a Pliance-X System (Novel GmbH, Munich, Germany). The pressure sensitive mat consisted of two separate parts each with 128 sensors in a 16 x 8 (longitudinal x transverse) array. Each sensor had a size of 4.7 x 3.1 cm (14.57 cm^2^). The sensor mat was calibrated prior to the experiment [7]. The two mat parts were placed symmetrically on each side of the horse’s back. Zero baseline was established before saddling and tightening the girth. Data were sampled at 70 Hz (four horses) or 60 Hz (three horses).

The horses were ridden on a high-speed treadmill (Mustang 2200) with an integrated force measuring system [12]. This system determines hoof positions during stance and decomposes the vertical ground reaction force responses at the multiple bearing points of the treadmill platform into four vertical hoof forces. Data were sampled at 420 Hz (five horses) or 480 Hz (two horses) [8].

Horse, rider and saddle movements were registered using multiple spherical reflective markers (19 mm ∅) attached with glue. Markers used were placed at the spinal processes of the sixth thoracic (T6), the third lumbar (L3), the third and fifth sacral vertebrae (S3, S5) and on the left and right tubera coxae of the horse. On the saddle they were placed on the caudomedial ends of the left and right saddle panels and the riders were equipped with markers on the sacrum and the left and right major trochanters of the femur. Marker positions were registered by 12 infrared optical cameras (ProReflex, Qualisys, Gothenburg, Sweden). Kinematic data were recorded at a sampling rate of 140 Hz for four horses and 240 Hz for three horses.

### Data analysis

Peak vertical ground reaction force (GRFmaxFore, GRFmaxHind) and vertical impulse (GRFimpFore, GRFimpHind) and temporal data for each limb were retrieved from the treadmill integrated force measuring system software [12]. Kinematic and saddle pressure data were exported to MATLAB (Matlab, The Math Works Inc., Natick, USA) for further processing and analysis.

**Fig 1:**
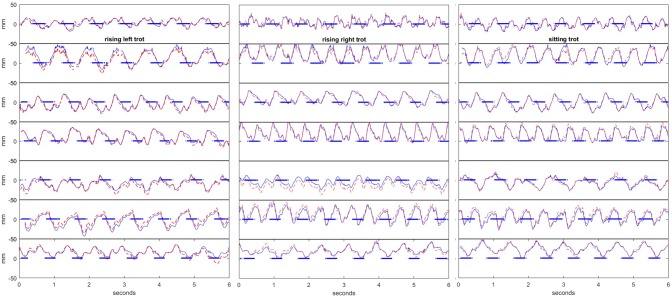
Lateral movement of the saddle. The blue line shows the lateral distance between the third lumbar vertebra and caudal part of the saddle perpendicular to the horse’s spine in all horses for left and right rising trot (rider rising on the left and right hind limb diagonal, respectively) and sitting trot. The dotted red line shows the same distance along the transverse axis of the laboratory coordinate system. Positive y-axis values indicate that the saddle is to the right of the vertebrae. The blue horizontal bars indicate right front/left hind limb diagonal supports.

Plots of raw marker coordinate data showed that the caudal part of the saddle moved laterally towards the supporting hindlimb during each diagonal step. To quantify this movement of the saddle relative to the horse's spine, the marker located at L3 and both markers located on the caudomedial ends of the saddle panels were used. The distance between the L3 marker and the mean of (midpoint between) the two saddle markers was calculated along a line orthogonal to a line between the L3 marker and the marker at T6 for each data frame (thereby correcting for deviation of the horse's spine from the longitudinal axis of the laboratory coordinate system, compare the two curves in Fig 1). The saddle-L3 distance curve was split into diagonal steps based on temporal information from the treadmill. A positive value was assigned for movement of the saddle to the supporting hindlimb’s side of the spine (i.e. to the left for left hind limb stance, and vice versa). Maximum (MAX) and positive area under the curve (AUC) were then determined for each diagonal step. AUC was calculated using the cumptrapz function in Matlab, which computes an approximation of the cumulative integral of the variable through the trapezoidal method with unit spacing.

Horse and rider pelvis segments were subjected to rigid body analysis by use of a previously published algorithm [13]. The rotations of each segment around the longitudinal, transverse and vertical axes were thereby described as three angles: roll, pitch and yaw, respectively. Zero rotation was defined as the position of the respective segments at square stance. The marker locations used to define the rigid body segments were for horse pelvis: the two tubera coxae and S3 and S5, and for the rider’s pelvis: the sacrum and the left and right major trochanters of femur.

Saddle pressure was recalculated to force and the total force over the entire mat was calculated for each data frame. Data for the rigid body rotations, the vertical excursion of markers located at T6 and S3, and total saddle force were split into strides based on ground contacts of the left hind limb, using temporal information from the treadmill, and normalized to 101 points (0–100%). From the stride normalized data, variables representing relevant extreme values (listed below) were extracted for each diagonal step. For bidirectional variables, positive direction was defined in relation to the hindlimb in stance phase, i.e. opposite directions for left and right hind limb diagonals. Roll and yaw rotations were defined as positive for rotation away from the supporting hindlimb side (i.e. clockwise rotation viewed from behind and from above, respectively, for the left hind limb diagonal). Pitch rotation was positive for clockwise rotation viewed from the right. The extracted variables were: maximum and minimum position of the T6 and S3 markers (MaxW, MinW, MaxP and MinP for withers and pelvis respectively); maximum roll and yaw rotation of the rider’s pelvis away from the supporting hind limb side; maximum roll rotation of the horse’s pelvis away from the supporting hind limb side; minimum yaw rotation of the horse’s pelvis towards (i.e. tail base moving away from) the supporting hind limb side; minimum and maximum pitch rotation of the horse’s pelvis; and maximum total saddle force.

### Statistics

The variable SEAT was constructed by categorizing diagonal steps as rider rising in rising trot (‘Rise’), rider sitting in rising trot (‘Sitrise’), or rider sitting in sitting trot (‘Sitsit’). The paired Wilcoxon signed rank test was used to compare horse means for the two saddle movement variables (MAX and AUC) between SEAT categories, as normality could not be verified in the small sample of mean values. Targeting the paired Student’s t-test, using a two-tailed alpha of 0.05, a power of 0.20, and a theoretical effect difference estimate of one unit, and the standard deviation of the difference slightly lower than the effect estimate, at 0.80 units (80% of the effect estimate), yields a sample size of five subjects. This was increased by two, to a final sample size of seven, to enable use of the non-parametric Wilcoxon matched pair test. Using this test on seven individuals statistical significance can be found if a variable in all seven horses has the same direction (p = 0.0156), if one horse deviates (p = 0.03) or if two horses deviate from the rest of the group (p = 0.04).

Descriptive statistics were calculated for the variables. For outcome data (MAX and AUC), normality was assumed based on normal quantile plots, with means and medians reasonably close, standard deviation reasonably small and values of skewness and kurtosis small. MAX was selected as outcome variable for multivariable analysis because its distribution was found to be reasonably normal. A mixed model was used (proc mixed in SAS, SAS Institute Inc. (2014) SAS/STAT 13.2 User’s Guide, SAS Institute Inc, Cary, North Carolina). The unit of observation was the diagonal step.

To model the random structure correctly a variable called horse-side was constructed (horse1- diagonal LF-RH, horse1- diagonal RF-LH, horse2- diagonal LF-RH etc). A priori including SEAT as a fixed effect, the random intercept structure contained horse, horse-side, trial number (1–21) to account for the clustering effect of diagonals belonging to the same measurement, and horse-side nested within trial number to account for the fact that the effects of left and right rising trot may differ in an individual horse due to the laterality/preferred side for that particular horse. The covariance structure was variance components in order to enhance convergence of models. This random structure was considered biologically relevant and produced the lowest Akaike criterion value.

Fixed effects in the models contained data on body weight of riders, saddle force (‘total saddle force’), kinetics (GRFimpFore, GRFimpHind, GRFmaxFore and GRFmaxHind), kinematics of the horses (MaxW, MinW, MaxP, MinP, ‘horse pelvis roll’, ‘horse pelvis pitch max’, ‘horse pelvis pitch min’ and ‘horse pelvis yaw’) and kinematics of the riders (‘rider pelvis roll’ and ‘rider pelvis yaw’). Preliminary models were made first, modelling each fixed effect separately (e.g. MaxW) on the full random structure and with SEAT included as fixed effect. Variables with p<0.05 were included in a full model, together with all possible two-way interactions with SEAT. This model was then reduced based on type III p-values and the Akaike criterion. In addition, models on only the sitting trot data (SEAT = ‘Sitsit’) were constructed, without the fixed effects SEAT variable, and neither with trial number or horse-side within trial number included in the random structure. Collinearity of fixed effect variables was investigated using Pearson correlation. MaxW, MinW, MaxP, MinP were expressed relative to horse mean (over all three trials) for the respective variables. Residual plots were scrutinised for normality/ heteroscedasticity. In addition, variables were also tested as squares in the dataset with rising and sitting trot (main effect and its square added upon the preliminary models) to evaluate whether they had a non-linear relationship with the outcome. Least square means and pairwise comparisons were computed if relevant. The p-value limit for statistical significance was set to 0.05.

## Results

Speed varied from to 3.19–3.42 m/s, mean speed for rising trials was 3.27 m/s and 3.26 m/s for sitting trials. The number of strides varied between 14 and 17 per trial, median 16. Fig 1 shows the distance between L3 and the saddle for all trials, before and after adjustment for the orientation of the horse's spine. S1 Fig shows examples of raw transverse axis coordinate data for the saddle and L3 for the three riding conditions (left rising, right rising and sitting) and S1 Table contains data used in the statistical analysis.

### Saddle symmetry variables

Descriptive statistics for AUC and MAX are shown in Table 1. Differences between SEAT categories were significant (p<0.05) except between sitting in rising trot and sitting in sitting trot (p = 0.11 for AUC, p = 0.08 for MAX). Overall, for the full data set, MAX had a mean of 19.5, SD 15.1, median 18.1, minimum -15.0 and maximum 56.4 mm. For the sitting trot data, the corresponding values were 19.7, 14.9, 19.8, -12.6 and 49.1 mm.

**Table 1:** Descriptive statistics of the saddle symmetry variables. Different superscripts (*, #) indicate significant differences, p<0.05, for the statistical comparison between SEAT categories (sitting or rising in rising trot, or sitting in sitting trot), using horse mean values.

SEAT	Variable	Mean	SD
Rise	AUC*	4.1	2.2
	MAX*	14.6	3.2
Sitrise	AUC^#^	7.4	2.2
	MAX^#^	24.1	6.1
Sitsit	AUC^#^	6.1	2.3
	MAX^#^	19.7	2.4

AUC area under the curve (mms) for lateral movement of the saddle relative to the horse’s spine towards the hind limb in support phase

MAX maximum lateral movement (mm) of the saddle relative to the horse’s spine towards the hind limb in support phase

### Models with rising and sitting trot

**Fig 2:**
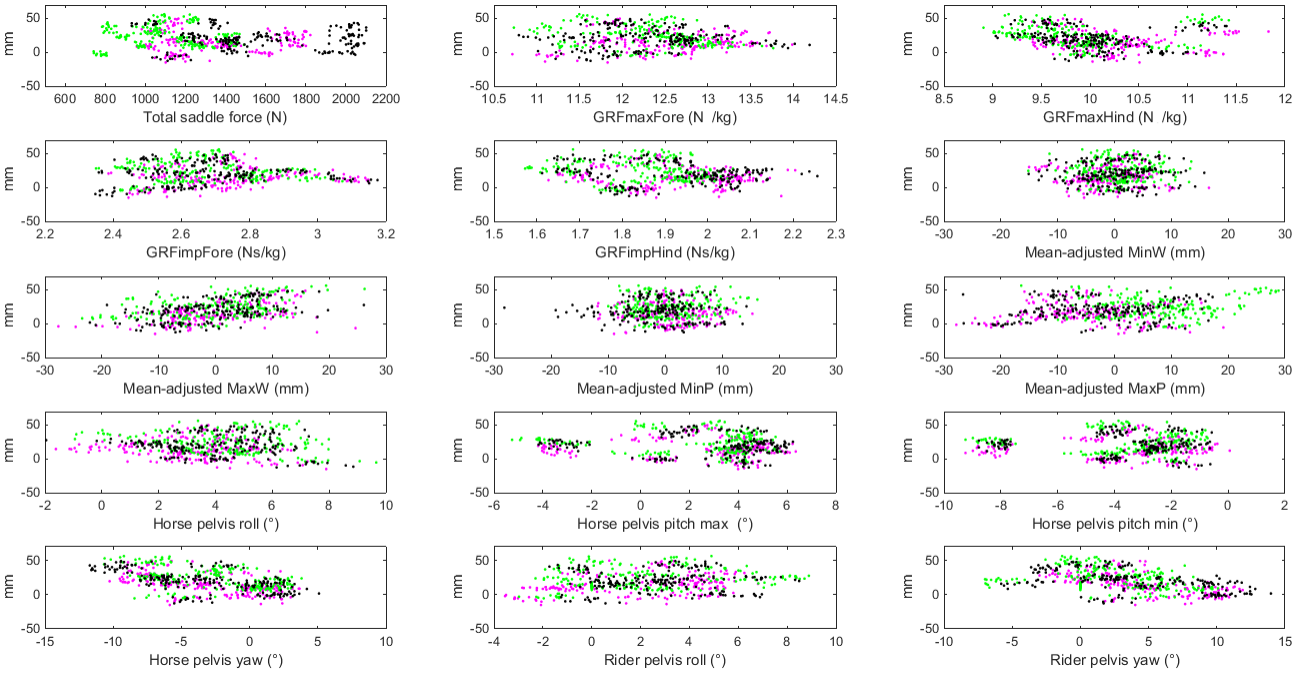
Evaluated fixed effect variables. Descriptive plots of independent fixed effect variables on the x-scale versus the outcome variable, lateral movement of the saddle in relation to the horse spine in mm, on the y-scale. Green is rising steps in rising trot, magenta is sitting steps in rising trot and black is sitting trot. The number of observations is 620, except for the rider kinematics where n = 590. Horse pelvis roll; positive rotation is away from the supporting hindlimb side Horse pelvis pitch; positive rotation is clockwise viewed from the right (i.e. tail up) Horse pelvis yaw; positive rotation is away from (i.e. tail towards) the supporting hindlimb side Rider pelvis roll; positive rotation is away from the supporting hindlimb side Rider pelvis yaw; positive rotation is away from the supporting hindlimb side.

Collinearity was found between the withers and pelvic vertical position variables (Pearson’s correlation coefficient, r>0.93), between horse pelvis pitch max and horse pelvis pitch min (r = 0.93) and horse pelvis yaw and rider pelvis yaw (r = 0.78). Fig 2 shows how all the modelled variables (except rider body mass) relate to MAX, by SEAT categories. There was marker loss for the rider’s pelvis in one trial (hence the number of observations varies between 620 and 590 diagonal steps).

Table 2 shows the preliminary models, including variables with p<0.20 for this dataset. Many of the ground reaction force variables and total saddle force were close to, though did not reach, statistical significance. If a confidence interval does not contain 1 then that category differs from the baseline category.

**Table 2:** Preliminary bi-variable mixed models with p-values <0.20. Rising and sitting trot data (620 diagonal steps, 21 trials, 7 horses except for in the two bottom models which are 590 steps, 20 trials, 7 horses) with the outcome variable, lateral movement of the saddle in relation to the horse spine, in mm. Bolded variables were entered into the full model for further reduction. Estimates and their 95% confidence intervals (95% CIs) are provided. Group P-values are used to evaluate significance of a variable as a whole (e.g. SEAT).

Variable	Category	Estimate	95% CI	Group P-value
**Intercept**		31.21	(19.78,42.63)	
** Total saddle force (N)**		-0.07	(-0.13,-0.02)	0.0067
** SEAT**	Rise	-6.73	(-9.52,-3.95)	<0.0001
	Sitrise	1.35	(-2.16,4.86)	
	Sitsit	BL^a^		
Intercept		15.59	(-1.65,32.83)	
GRFmaxFore (N/kg)		0.33	(-0.92,1.58)	0.6028
SEAT	Rise	-5.39	(-8.11,-2.68)	<0.0001
	Sitrise	4.63	(1.92,7.33)	
	Sitsit	BL		
Intercept		7.88	(-9.91,25.67)	
GRFimpFore (Ns/kg)		4.40	(-1.57,10.38)	0.1492
SEAT	Rise	-5.52	(-8.26,-2.77)	<0.0001
	Sitrise	4.70	(1.97,7.44)	
	Sitsit	BL		
**Intercept**		19.77	(11.98,27.57)	
** MinP (mm)**		0.08	(0.02,0.15)	0.0136
** SEAT**	Rise	-5.49	(-8.22,-2.77)	<0.0001
	Sitrise	4.49	(1.77,7.21)	
	Sitsit	BL		
**Intercept**		20.13	(12.32,27.94)	
** MaxP (mm)**		0.15	(0.09,0.20)	<0.0001
** SEAT**	Rise	-5.11	(-7.86,-2.35)	<0.0001
	Sitrise	3.02	(0.20,5.85)	
	Sitsit	BL		
**Intercept**		21.06	(13.20,28.91)	
**Horse pelvis roll (◦)**^b^		0.35	(0.01,0.69)	0.045
** SEAT**	Rise	-5.57	(-8.26,-2.88)	<0.0001
	Sitrise	4.77	(2.08,7.45)	
	Sitsit	BL		
Intercept		20.95	(13.05,28.86)	
Horse pelvis pitch max (◦)^c^		-0.47	(-1.00,0.07)	0.0869
SEAT	Rise	-5.44	(-8.16,-2.73)	<0.0001
	Sitrise	4.42	(1.70,7.14)	
	Sitsit	BL		
Intercept		20.28	(12.23,28.33)	
Horse pelvis yaw (◦)^d^		0.19	(-0.08,0.46)	0.162
SEAT	Rise	-5.34	(-8.04,-2.65)	<0.0001
	Sitrise	4.58	(1.89,7.28)	0
	Sitsit	BL		
**Intercept**		23.25	(14.87,31.64)	
**Rider pelvis roll (◦)**^e^		1.31	(0.99,1.64)	<0.0001
** SEAT**	Rise	-6.46	(-8.69,-4.23)	<0.0001
	Sitrise	4.36	(2.13,6.59)	
	Sitsit	BL		
**Intercept**		16.59	(7.75,25.42)	
**Rider pelvis yaw (◦)**^f^		0.78	(0.56,1.00)	<0.0001
** SEAT**	Rise	-5.63	(-8.34,-2.92)	<0.0001
	Sitrise	5.82	(3.10,8.53)	
	Sitsit	BL		

^a^BL- baseline

^b^Horse pelvis roll; positive for rotation away from the supporting hindlimb side

^c^Horse pelvis pitch; positive for clockwise rotation viewed from the right (i.e.tail base moving upwards)

^d^Horse pelvis yaw; positive for rotation away from (i.e. tail base moving towards) the supporting hindlimb side

^e^Rider pelvis roll; positive for rotation away from the supporting hindlimb side

^f^Rider pelvis yaw; positive for rotation away from the supporting hindlimb side

Table 3 shows the final multivariable model for this dataset. None of the variables in the preliminary models had significant squares. The last variable excluded from the model had p = 0.14 (the interaction between SEAT and rider pelvis roll). Final residual plots were considered adequate. Least square means by SEAT were 14.3 (SE 4.7) mm for rising in rising trot, 23.9 (SE 4.7) mm for sitting in rising trot and 20.3 (SE 4.7) mm for sitting in sitting trot. The comparison between sitting in rising trot and sitting in sitting trot was associated with p = 0.004 and the other two comparisons with p<0.0001, in line with the univariable non-parametric comparison in Table 1.

**Table 3:** Multivariable mixed models. Model results for the outcome lateral movement of the saddle in relation to the horse spine in mm, for rising and sitting trot data (590 diagonals, 20 trials, 7 horses) and from sitting trot data (208 diagonals, 7 trials, 7 horses). Estimates and their 95% confidence intervals (95% CIs) are provided.

Rising and sitting trot (n = 590)	Category	Estimate	95% CI
Intercept		20.47	(11.21,29.72)
SEAT	Rise	-6.07	(-8.42,-3.73)
	Sit	3.55	(1.13,5.97)
	SitSit	BL^a^	
MaxP (mm)		0.14	(0.08,0.19)
Rider pelvis roll (◦)^b^		-1.14	(-1.45,-0.83)
Rider pelvis yaw (◦)^c^		0.69	(0.49,0.90)
Sitting trot model (n = 208)			
Intercept		20.48	(10.85,30.11)
MaxP (mm)		0.22	(0.13,0.31)
Rider pelvis roll (◦)		-1.34	(-1.91,-0.77)
Rider pelvis yaw (◦)		0.89	(0.56,1.23)

^a^ BL- baseline

^b^ Rider pelvis roll; positive for rotation away from the supporting hindlimb side

^c^ Rider pelvis yaw; positive for rotation away from the supporting hindlimb side

Maximum pelvic height at push off had a numerically small but still highly significant effect on the saddle movement. A MaxP increase of 10 mm produced an added 1.4 mm of lateral saddle movement towards the supporting hind limb (MAX).

Rider pelvis roll had almost twice the effect compared to rider pelvis yaw, per degree of motion, and the rotations had an opposite influence on the saddle movement. For each degree extra of roll away from the supporting hind limb, MAX decreased with 1.1 mm and for each degree extra of yaw rotation away from the supporting hind limb, MAX increased with 0.7 mm. From the graphs in Fig 2 we see that these variables span 15 degrees (rider pelvis roll) and 25 degrees (rider pelvis yaw). As an example, if roll of the rider's pelvis away from the supporting hind limb increased with 5 degrees, this is associated with a relatively substantial decrease in lateral saddle movement (MAX) of 6 mm.

### The model with sitting trot only

Collinearity between the 16 variables was found for all the withers and pelvic vertical position variables (r>0.93), between horse pelvis pitch max and horse pelvis pitch min (r = 0.94), between horse pelvis yaw and rider pelvis yaw (r = 0.86), between GRFimpFore and GRFimpHind (r = 0.70) and between rider body weight and total saddle force (r = 0.82). The black dots in Fig 2 show data for sitting trot.

Table 3 shows the final model and Table 4 the preliminary analyses. The last variable that was excluded from the model was horse pelvis pitch (at p = 0.06). Residual plots were considered satisfactory.

**Table 4:** Preliminary univariable sitting trot mixed models with p-values <0.20. Sitting trot data (208 diagonals, 7 trials, 7 horses) with the outcome variable, lateral movement of the saddle in relation to the horse spine, in mm. Bolded variables were entered into the full model for further reduction. Estimates, their 95% confidence intervals (CIs) and Walds P-values are provided.

Variable	Estimate	95% CI	Walds P-value
**Intercept**	34.08	(18.57,49.58)	**0.01**
** Total saddle force**	-0.01	-(0.02,0.00)	**0.03**
Intercept	0.92	-(24.43,26.28)	0.95
GRFmaxFore (N/kg)	1.53	-(0.43,3.49)	0.13
Intercept	38.50	(10.98,66.02)	0.03
GRFmaxHind (N/kg)	-1.88	-(4.52,0.75)	0.16
Intercept	-3.92	-(29.27,21.43)	0.77
GRFimpFore (Ns/kg)	8.81	-(0.18,17.81)	0.06
Intercept	36.92	(14.97,58.86)	0.02
GRFimpHind (Ns/kg)	-9.02	-(19.74,1.70)	0.10
**Intercept**	20.23	(12.31,28.15)	**0.002**
** MaxP (mm)**	0.18	(0.08,0.28)	**0.001**
**Intercept**	22.76	(14.44,31.09)	**0.002**
**Horse pelvis pitch max (◦)**^a^	-1.12	-(2.02-,0.22)	**0.02**
**Intercept**	22.58	(14.27,30.89)	**0.002**
**Rider pelvis roll (◦)**^b^	1.06	(0.44,1.69)	**0.001**
**Intercept**	17.05	(8.06,26.03)	**0.01**
**Rider pelvis yaw (◦)**^c^	0.66	(0.30,1.02)	**0.0004**

^a^Horse pelvis pitch; positive for clockwise rotation viewed from the right (i.e. tail up)

^b^Rider pelvis roll; positive for rotation away from the supporting hindlimb side

^c^Rider pelvis yaw; positive for rotation away from the supporting hindlimb side

The conclusions from this model are that the estimates for rider pelvis roll and yaw are very similar to the full model, and that MaxP has a somewhat larger effect compared to the model that includes data on rising trot (Table 3).

## Discussion

Previous studies have found an association between progressive lateral displacement of the saddle during riding, termed saddle slip, and lameness and crooked/asymmetric riders, based on subjective evaluation [1–3]. In this study we used objective measures of oscillations of the saddle with high temporal resolution and multivariable models to evaluate associations between horse and rider movements and the magnitude of lateral saddle movement during the stride cycle in well-ridden, non-lame horses without evidence of clinically relevant saddle slip. Saddle movement was assessed based on the position of the caudal part of the saddle, illustrated by photos taken from behind [1]. In this study we used markers placed at the caudal part of the saddle and at L3 of the horse, and measured the distance between them in right angles to the horse’s dorsal midline. The maximum distance, representing maximum lateral movement of the saddle towards the weight-bearing hindlimb side during each diagonal step, was used as outcome variable in multivariable models. Considering the explanatory variables that remained in the final model, three of the four variables related to the rider’s movements were significant (SEAT and roll and yaw of the rider’s pelvis, but not maximum saddle force), whereas only one of the many variables related to the horse's movements was retained. The estimates for the different SEAT categories showed that the lateral movement of the saddle was clearly smaller for rising steps compared to both sitting steps in rising trot and sitting trot. The greatest movement occurred when the rider sat down on the saddle after a rising step. This means that the saddle moves asymmetrically during rising trot, with a larger movement towards the hindlimb of the sitting diagonal and a smaller movement towards the hindlimb of the rising diagonal. It has been shown in previous studies that rising trot induces a significant asymmetry to the horse's movements [10–11], and it is therefore not surprising that the saddle too moves asymmetrically (relative to the horse) during rising trot. The rising trot example shows that horse or rider movement asymmetries can result in asymmetric saddle movement, which, in turn, could be the underlying mechanism behind saddle slip.

In association with lameness, saddle slip has been found to occur towards the side of the lamer hindlimb in a majority of cases [1]. Whether and how the dynamic non-progressive saddle motion we identified was associated with the weight-bearing hindlimb must therefore be considered. In this study, a lower pelvic vertical position after push-off was associated with a decrease in the lateral movement of the saddle towards this hind limb’s side. Since there was no interaction between MaxP and SEAT, this effect was consistent regardless if the rider was sitting or rising at the trot. Hindlimb lameness has been associated with a lower pelvic vertical position after push-off of the lame hindlimb [14–15]. Consequently, our results may seem contradictory to the previous observational studies. However, the relative influence of the vertical movement of the pelvis was minor, based on the model estimates (Table 3), compared to other factors, such as the rider. Therefore, the overall effect of an asymmetric movement pattern may be different from the specific influence of the upwards movement of the pelvis. A lower pelvic position after push-off has been shown to reflect a redirection of force with decreased vertical impulse during the second half of hindlimb stance and increased horizontal propulsive impulse [14]. This could result in the rider's ipsilateral hip being pushed more forward and less upward, increasing yaw rotation and decreasing roll rotation of the rider's pelvis. This, in turn, would increase the lateral movement of the saddle towards the lame hindlimb. In previous observational studies, it was found that the likelihood of saddle slip in lameness decreased with a heavier rider, whereas the opposite was true for asymmetric saddles [1]. This supports the idea that the horse-rider-saddle interaction is of more importance than horse or rider movements on their own. In this study, a negative association was found between maximum total saddle force (not normalized to rider mass) and maximum lateral movement of the saddle (Tables 2 and 4). Also, in the preliminary models (Tables 2 and 4) increased lateral saddle movement was associated with increased pelvic vertical position at midstance (minP), and decreased hind limb vertical peak force and impulse, all of which has been observed in hind limb lameness [14].

Since saddle slip has mainly been visually evaluated [1–3] it is relevant to consider whether the saddle motion evaluated in this study could be visually detected. The range for the lateral movement of the caudal part of the saddle relative to L3 was only about 2 cm on average (Table 1). The lateral movement of L3 is typically of about the same range [16], but can be up to four times larger in individual horses [17]. Considering this and the numeric value of the estimates (Table 3), it seems very difficult to evaluate asymmetries of the lateral movements of the saddle by unaided visual observation, or from plain videos. Viewing the saddle of a large horse from the ground, and/or viewing the movement of the saddle from the side of the horse, makes it even more difficult due to projection effects. For a clinical subjective assessment to be reliable, it is likely necessary that the asymmetry is large enough to result in a progressive lateral displacement, slipping, of the saddle. Also, in previous observational studies saddle slip was evaluated and graded over an entire ride [1–2].

The effect of the rider’s pelvic motion on the lateral movement of the saddle seems to be determined by the relative proportion between rotation around the vertical and the longitudinal axis, respectively, since yaw rotation increased the lateral saddle movement whereas roll rotation decreased the movement. This was true both for sitting and for rising trot. From Fig 1 it is visually obvious that each horse and rider have their own typical pattern of lateral saddle movement. The individual patterns are consistent between trials, and the inter-individual differences appear larger than the group level effect of sitting or rising trot. Unique interaction between rider and horse in each couple is likely the cause. The rather strong correlations between many of the explanatory variables indicate that overall systematic individual patterns were present in the data. During the statistical analysis, it was obvious that finding the correct random structure was crucial for a sound model. It was also noted that the lateral movement of the saddle (Fig 1), as well as several other bidirectional variables, had an offset, with horse/rider mean values deviating from zero. This may partly be due to experimental artifacts, such as asymmetric placement of markers, but is likely also reflecting laterality of the individual horses and/or riders. It was therefore crucial to include not only horse, but also horseside, as random factor in the models.

To investigate the movements of the saddle relative to the horse in full detail, it would have been ideal to perform 6DOF calculations using rigid body models of the saddle and the horse's trunk. Unfortunately, this was not possible with the marker setup used in the current study. However, to assess the relative influence of rotation of the saddle about the longitudinal axis (roll), we determined the range for the vertical distance between the two saddle markers (as a proxy for roll). Results from trigonometry calculations suggested that this rotational movement caused a lateral movement of the saddle that was less than 10% of the maximum distance measured between the saddle markers and L3 of the horse, in all measurements. We therefore concluded that the distance used as outcome in this study mainly represents rotation of the saddle about the vertical axis (yaw) and/or lateral translation of the saddle. We are, however, not able to distinguish between the latter two types of movement.

In this study, the lateral movement of the saddle was related to the movements of both the horse and the rider. Still, this study did not evaluate all possible factors that could influence the saddle’s movement. Saddle slip, typically visually assessed comparing saddle position at the start and the end of the ride, has been associated with conditions not evaluated in this study, such as asymmetric saddles. Saddle type and construction details, such as girthing, may also influence the range of lateral movement. Girthing has been shown to affect the saddle pressure pattern [5]. The major limitation of this study was that only seven horses were included, which limits extrapolation to the general horse population. It also limits the power of the study for finding all biological determinants for lateral saddle movements. Furthermore, all horses were warmbloods bred and used for dressage, which are likely to have more extravagant movements than the general riding horse. Another limitation of this study is that saddle movements were only measured in steady-state straight line trot on a treadmill. In observational studies on saddle slip the horses were evaluated ridden overground, both on the straight and on circles, and at both trot and canter. Saddle slip in horses with hindlimb lameness was usually worse on the circle compared with the straight [1–2]. It is also possible that certain exercises, such as rising trot or transitions, make the saddle more prone to slip. Further studies should investigate the biomechanics behind saddle slips in greater detail and under more field-like conditions.

### Conclusion

The rider exerts a large influence on the lateral movement of the saddle. Since the rider’s movements are closely coupled to the movements of the horse [4], we can, however, not conclude that the rider is a more important factor compared to the horse. Nevertheless, we found that it is of importance how the rider reacts to the horse's movements. The fact that many linear relationships were found between the lateral movement of the saddle and movements of the horse and rider implies a relationship between saddle and horse and/or rider movement asymmetries. The ranges and effect sizes suggest that it is challenging for the unaided observer to evaluate this, unless the saddle lateral movement asymmetry is large enough to result in a progressive lateral displacement of the saddle during the ride.

## Supporting information

S1 Fig Example of raw data. Lateral movement of the saddle and L3 in one horse for left rising, right rising and sitting trot. Higher y-axis values imply movement to the right and lower values movement to the left.
(TIF)

S1 Table Data used for analysis. (XLSX)
