# Supplementary figures and images for: Lateral movement of the saddle relative to the equine spine in rising and sitting trot on a treadmill
Source: PLoS One. 2018 Jul 18;13(7):e0200534. doi: 10.1371/journal.pone.0200534 (PMC6051618; doi:10.1371/journal.pone.0200534)

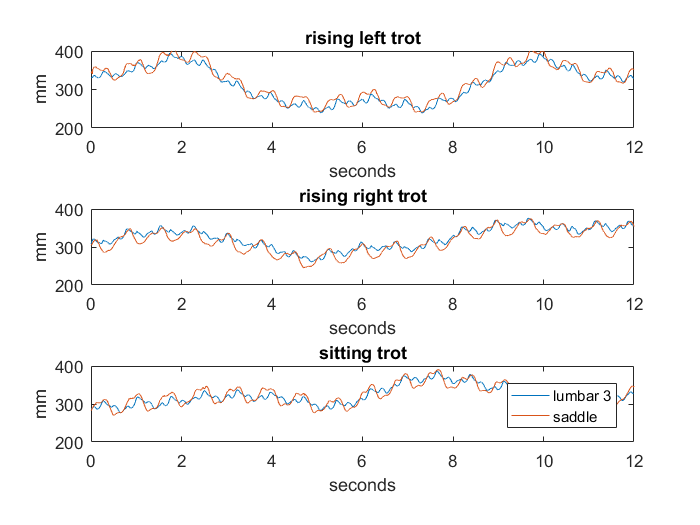

Supplement: S1 Fig — Lateral movement of the saddle and L3 in one horse for left rising, right rising and sitting trot. Higher y-axis values imply movement to the right and lower values movement to the left. (TIF) [file pone.0200534.s001.tif]
